# Optimizing Motion Management and Baseline Shifts in Magnetic Resonance-Guided Spine Stereotactic Body Radiation Therapy

**DOI:** 10.3390/cancers17162697

**Published:** 2025-08-19

**Authors:** Yao Ding, Travis C. Salzillo, Debra N. Yeboa, Martin C. Tom, Zhiheng Wang, Parmeswaran Diagaradjane, Ergys Subashi, Jinzhong Yang, Todd Swanson, Thomas Beckham, Chenyang Wang, Amol J. Ghia, Tina Briere, Jihong Wang, Fabienne Lathuilière, Sneha Cloake, Eun Young Han

**Affiliations:** 1 Department of Radiation Physics, The University of Texas MD Anderson Cancer Center, Houston, TX 77030, USA; yding1@mdanderson.org (Y.D.); ehan@mdanderson.org (E.Y.H.); 2 Department of Radiation Oncology, The University of Texas MD Anderson Cancer Center, Houston, TX 77030, USA; 3 Elekta AB, 103 93 Stockholm, Sweden

**Keywords:** spine SBRT, MR-guided radiation therapy, motion management

## Abstract

Background: Stereotactic body radiation therapy (SBRT) has proven effective in controlling spinal lesions with minimal toxicity, primarily due to its ability to limit spinal cord dose. Recent advances in MR-linac (MRL) technology offer superior spinal cord visualization and real-time gating, which can facilitate dose escalation in spinal tumor treatment while maintaining safety. Purpose: This study aimed to optimize motion management for spine SBRT on an MRL by analyzing patient-specific motion dynamics and evaluating the most effective registration structures. We hypothesized that baseline shifts (BLS) would improve delivery efficiency while maintaining spinal cord dose constraints. The goal was to establish displacement thresholds and assess the role of baseline shift correction adaptative planning in improving treatment delivery efficiency. Methods: Twelve patients underwent two MRI sessions on the MRL. The optimal registration structure was identified, and intrafraction motion was assessed to calculate delivery efficiency. Baseline shift (BLS) simulations were applied for five cases that showed significant motion and suboptimal delivery efficiency, and the dosimetric impact of the BLS was evaluated. The simulated BLS-based plan adaptation was implemented via a segment aperture morphing adapt-to-position workflow. Results: The most stable registration structure was the spinal canal plus three adjacent vertebrae. Cine imaging revealed average intrafraction motion (95th to 5th percentiles) of 0.8 ± 0.5 mm in the right-left (RL) direction, 0.9 ± 0.6 mm in the anterior–posterior (AP) direction, and 0.7 ± 0.5 mm in the SI direction. Simulated BLS improved delivery efficiency to >80% in all but one case, with a ±1 mm displacement threshold tolerance. While target coverage remained consistent after BLS simulation, the spinal cord dose increased by 7–60%, exceeding the 14 Gy constraint in three of the five simulated cases. Conclusions: Cine imaging and BLS can enhance delivery efficiency in spine SBRT but may increase spinal cord dose. These findings underscore the need for careful patient selection, advanced motion management, and patient-specific BLS protocols.

## Simple Summary

This imaging-only study investigates the capabilities of a new motion management system on the MR-Linac for treating spine stereotactic body radiation therapy (SBRT) patients. Three registration structures were tested for tracking accuracy, and intrafraction motion was measured to propose gating tolerances. Sessions with low delivery efficiency were subjected to simulated baseline shifts (BLS), which resulted in significantly improved delivery efficiency. Plan adaptation based on BLS resulted in significantly higher spinal cord max doses in some cases, though no significant differences in target coverage or minimum dose were observed. These findings highlight both the benefits and cautions required when applying BLS in spine SBRT.

## 1. Introduction

Stereotactic body radiation therapy (SBRT) has developed into a mainstay treatment for secondary metastases to the vertebral column with the goal of delivering a cytotoxic dose to the lesion while preserving the radiosensitive spinal cord [1,2,3,4]. One-year local control rates have improved to as high as 90% following aggressive single- and hypo-fractionated dose regimens, while local failures have been attributed to incomplete coverage of lesions with 15 Gy [5,6,7,8,9]. Even the local control of high-Bilsky-grade tumors increased following hybrid therapy (separation surgery and concomitant spine SBRT) due to the additional coverage [10]. Thus, if sufficient care is taken to minimize spinal cord injury, continued efforts to increase the biological effective dose (BED) and coverage to these lesions should lead to more durable outcomes.

Fortunately, recent studies have demonstrated that single-fraction cord doses up to 14 Gy were associated with less than 1% chance of radiation myelopathy, which suggests that the conventional 12 Gy constraint could potentially be loosened [11,12,13]. It should be noted that none of these studies utilized a planning organ at risk volume (PRV) expansion for the cord. However, there is evidence that arterial and craniospinal fluid pulsations could lead to cord motion of up to 1.5 mm within the thecal sac and corresponding cord dose increases of 10–20% [14,15].

Because X-ray-based onboard imaging in conventional linacs lacks the soft-tissue contrast to visualize the spinal cord or monitor intrafraction motion, there is understandable hesitation to escalate doses without a PRV expansion, which may limit target coverage. In contrast, MR-guided approaches offer real-time imaging of both target and critical structures, increasing the confidence of dose delivery. Therefore, there is rationale to commission spine SBRT treatments on a hybrid MR/linac device (MRL). The MRL can acquire various types of image contrast to visualize the target and surrounding tissues, including dynamic cine scans, which can acquire orthogonal, single-slice images at a frequency of 5–10 frames per second, including while the radiation beam is on [16,17,18,19]. Dosimetry on these accelerators has been well-characterized, and the feasibility of treating spine metastases has been demonstrated [20,21,22].

A major upgrade, known as Comprehensive Motion Management (CMM), was recently installed on the MRL (Marlin version 5.7) at our institution and allows for automatic target tracking and gating of the beam when significant motion is detected in the cine MR images [23,24,25]. It is also capable of fast, intrafraction plan adaptation, known as baseline shift (BLS), to account for non-periodic motion that would otherwise result in excessive or continuous beam holds [26,27]. Rusu et al. describe gating strategies and how they are incorporated within the clinical MR-guided radiotherapy workflow [28]. Conceivably, one can use this technology to track the spinal cord in real-time and gate the beam when it enters a sufficiently high-dose region, allowing for precise dose control to the cord. Unlike prior studies relying on onboard X-ray-based systems or studies that investigated cord motion during MR simulation, this study provides novel data on real-time cord tracking, beam gating, and plan adaptation using clinical tools and setup that are available on the MRL for spine SBRT, which addresses a critical gap in the motion management literature.

The goal of our pilot study was to characterize key aspects of MRL gating in the context of spine SBRT prior to its clinical implementation. Specifically, we aimed to optimize template structures for tracking target and spinal cord motion, quantify the amount of motion in these structures, and investigate the impact of BLS plan adaptation on delivery efficiency and dosimetry. This analysis was performed on 3D T2-weighted MR and cine MR images acquired from consenting patient volunteers in the treatment position on the MRL. We hypothesized that BLS would improve delivery efficiency while maintaining the spinal cord dose within acceptable constraints. While previous MR-Linac studies focused on feasibility or dosimetry in spine SBRT, this is the first to evaluate motion tracking accuracy and BLS strategies in a systematic imaging-only study [29].

## 2. Materials and Methods

### 2.1. Patients

Twelve patients were enrolled in an IRB approved prospective clinical protocol (PA22-0589) from July 2023 through May 2024. In addition to their standard workup for spine SBRT, they participated in two MR simulations at the MRL: one following their CT simulation and the second prior to their radiation treatment (Table 1). Patients were immobilized on an SBRT board with adjustable foot and leg rests, knee compression bridge, respiratory belt, and head cushion (Klarity Medical, Guangzhou, China). Five-point thermoplastic masks (Orfit Industries, Wijnegem, Belgium) were used for immobilizing patients treated for cervical spine (C-spine) and upper thoracic spine (T-spine) lesions, and a vacuum bag cradle overlaid on a wing board (Klarity Medical, Guangzhou, China) was used for immobilizing patients with lower T-spine lesions. The sample size (*n* = 12) was based on feasibility and the exploratory nature of this pilot study.

### 2.2. MRI Motion Data Acquisition and Data Processing

MR images were acquired with a Unity MRL system (Elekta AB, Stockholm, Sweden), which is based on a 1.5 T MRI manufactured by Philips (Philips Healthcare, Best, The Netherlands). The scan protocol included a 3D T2 MR scan (turbo spin echo, T2 weighting, TR/TE: 2100/375 ms, pixel bandwidth: 459 Hz, field-of-view: 500 × 500 mm^2^, reconstruction voxel size: 0.68 × 0.68 × 1.1 mm^3^), followed by 20 min of 2D interleaved sagittal and coronal cine imaging (balanced turbo field echo, T2/T1 weighting, TR/TE: 4.6/2.3 ms, pixel bandwidth: 478 Hz, field-of-view: 500 × 500 mm^2^, reconstruction voxel size: 0.96 × 0.96 × 5 mm^3^, frame rate: 1.2 fps per slice orientation), and concluded with a repeated 3D T2 MR scan. Images were obtained with a vendor-supplied body coil and quadrature surface coil.

Intrafraction motion was assessed in two ways from these images. The first was via registration of the 3D T2 images that were acquired pre- and post-cine imaging. The second approach analyzed cine MR images with the Elekta Motion Management Research Package (MMRP) software (version 4.5). This required the user to define a registration structure within the image to track. The output data consists of a spreadsheet detailing the registration structure centroid position, 3D displacement from the initial position, and average displacement during each cine acquisition time point. The difference between the maximum and minimum position of the registration structure in each plane (Max–Min) was calculated. To filter out large displacements caused by sudden movements such as coughing or by tracking errors, we also calculated the range between the 95th and 5th percentiles (95%–5%) of the registration structure’s position values [30]. The average range of motion within a 95% confidence interval was calculated as the mean plus 2 standard deviations of the (95%–5%) position values and was used as the displacement threshold for calculating delivery efficiency, which is described in the following sections.

### 2.3. Optimization and Motion Analysis of Registration Structures

**Figure 1:**
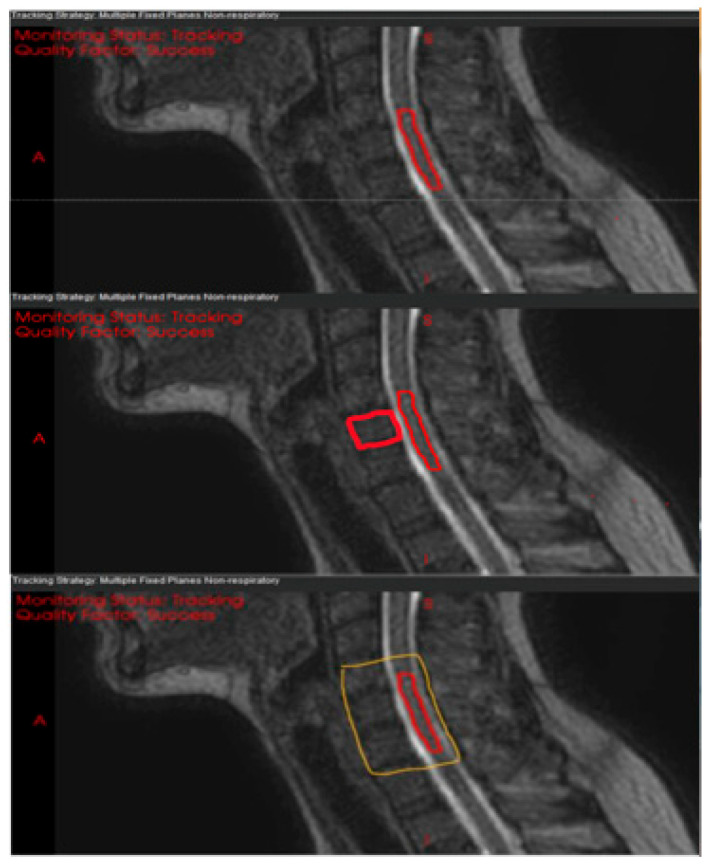
Three candidate registration structures for motion tracking on sagittal MRI scans: (**top**), spinal cord only; (**middle**), clinical target volume (CTV) plus cord; (**bottom**), spinal canal plus three adjacent vertebrae.

Next, we sought to identify the most stable registration structure to use for motion management by exploring options based on the spinal cord with the MMRP template tracking algorithm. We tested three candidate registration structures: the spinal cord alone; the clinical target volume (CTV) + cord; and three adjacent vertebrae plus the spinal canal (canal + 3VB) (Figure 1). The calculated motion was compared with visual assessment of the motion to determine the tracking accuracy and consistency between registration structures. To support these findings, we also calculated the range of motion (Max–Min) of the structures from 3 patient cases. Once the optimal registration structure was determined, its range of motion (95%–5%) was calculated across all cases and compared with the intrafraction motion measured between the two 3D MRIs.

### 2.4. Delivery Efficiency and Baseline Shift Correction

The MMRP output file was used to calculate the displacement of the registration structure throughout the cine acquisition relative to its initial position. Delivery efficiency was calculated for each case as the proportion of time that the position of the registration structure was within the displacement threshold (as calculated above), divided by the total time of the cine acquisition. This is equivalent to what is reported on the online CMM interface.

For cases in which delivery efficiency was low (70% or lower), we simulated the effect of a BLS to improve the efficiency. Instead of calculating displacement relative to the initial position for the entire cine acquisition, a time-varying baseline position was used to determine the registration structure’s displacement. The initial position of the baseline was equal to the initial position of the registration structure and remained constant until certain criteria were met. These criteria were applied consistently to each patient’s motion data. The rationale for the following criteria was based on internal pilot data and discussions with clinical users to reflect realistic scenarios for implementing baseline shifts.

- More than 1 min had passed since the start of the cine acquisition or the last baseline correction;
- Calculated delivery efficiency was below 80% for the prior minute;
- More than 10 s remained in the cine acquisition session.

If these conditions were met at any point during the cine acquisition, the baseline position was updated to the average registration structure position at that time point, as reported by the MMRP software. All subsequent registration structure displacement values in the cine acquisition were then calculated relative to the updated baseline value until either a new baseline was established by the above criteria or the cine acquisition ended. This effectively replicated the displacement calculations in the CMM software when a clinical BLS is implemented. The updated registration structure displacement values were then used to calculate BLS delivery efficiencies.

In addition to changes in delivery efficiency following a BLS, we also sought to understand associated dosimetric changes. BLS plan adaptation is essentially a global shift of the multileaf collimator control points to match the new registration structure position. It is equivalent to segment aperture morphing (SAM) in the adapt-to-position (ATP) workflow [27]. Only the five patients with delivery efficiency < 70% due to significant motion were included in the BLS dosimetry analysis to represent clinically relevant scenarios where BLS would be considered. For these patients (Patients 3, 4, 6, 7, and 9), we created a reference plan from the first (pre-cine) 3D T2 image of the MR simulation session and performed a SAM-based ATP using the second 3D T2 image (post-cine), without additional optimization, to represent an adapted BLS plan. This assumes that the second 3D MR image represents the mid-treatment target position that could have triggered a BLS during a clinical treatment. From this ATP, we calculated changes in dose and volumetric coverage to the GTV, CTV, and cord. The plans were normalized based on the reference plan GTV coverage, and a cord dose constraint of D_0.01cc_ ≤ 14 Gy was applied for the simulated BLS plan.

### 2.5. Statistical Analysis

Delivery efficiency, CTV V_100%_, and GTV V_100%_ percentage values were converted to decimal format. To expand the range beyond [0, 1] and lower the bias of parametric statistical tests, these distributions were transformed using the logit transformation [31]. For these transformed distributions and the cord max dose and GTV min dose, normality/lognormality was tested using the Shapiro–Wilk test. If the paired distributions were both normal, a two-tailed paired *t*-test was used to test for significant differences. If the paired distributions were both lognormal, then a two-tailed ratio paired *t*-test was used to test for significant differences. If one or both of the distributions were non-(log)normal, then a two-tailed non-parametric Wilcoxon matched-pairs signed-rank test was used to test for significant differences. We used a one-sided statistical test for the delivery efficiency because BLS simulations necessarily have a non-reductive effect on these values. Significant differences were attributed to comparisons with *p* < 0.05.

## 3. Results

### 3.1. Optimization of the Registration Structure

Based on visual assessment of the MMRP template tracking, the ‘cord only’ and ‘cord + CTV’ structures were prone to inaccuracies and showed inconsistent motion tracking due to structural similarities with adjacent cord and vertebrae. The ranges of motion (Max–Min) measured from cine images for the three patients used in the registration structure optimization were consistent within 0.4 mm across the different registration structures, except in the superior–inferior (SI) direction, where the cord-only structure showed discrepancies of up to 3.0 mm compared to the other registration structures (Table 2). This is likely due to the small size and indistinct boundaries of the spinal cord in that direction. When the CTV and adjacent three vertebrae were included in the registration structure, SI motion tracking seemed to be more consistent across patients and tended to exhibit smaller variations. Therefore, for the remaining analyses, the “canal + 3VB” registration structure was used.

### 3.2. Range of Intrafraction Motion

The intrafraction motion (95%–5%) of the registration structure, measured by real-time cine imaging across all patients was 0.8 ± 0.5 mm (mean ± standard deviation) in the right-left (RL) direction, 0.9 ± 0.6 mm in the anterior–posterior (AP) direction, and 0.7 ± 0.5 mm in the superior–inferior (SI) direction. In comparison, intrafraction motion assessed using the two 3D MRIs yielded values of 0.6 ± 0.4 mm in the RL direction, 0.6 ± 0.7 mm in the AP direction, and 0.6 ± 0.3 mm in the SI direction. Thus, motion calculated from real-time cine imaging was, on average, 0.2–0.3 mm greater than that estimated from the pre- and post-cine 3D MRI scans. While these comparisons were not statistically significant (*p* > 0.05), they demonstrate that periodic static imaging may not capture full target or OAR motion during beam delivery.

### 3.3. Delivery Efficiency

**Figure 2:**
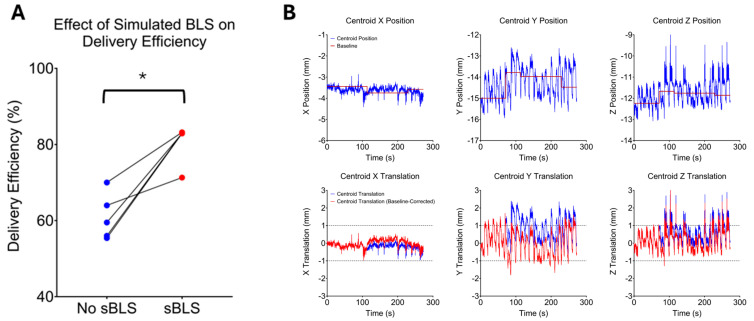
Effects of simulated baseline shifts (sBLS) on delivery efficiency. Average delivery efficiency was significantly improved following simulated BLS in the 5 patients who had initial delivery efficiencies < 70% (**A**). Real-time positions (**top row**) and displacements (**bottom row**) of the registration structure for Patient 3 (**B**). The top row also illustrates the simulated baseline (red line) of the registration structure. The bottom row illustrates the resultant displacement of the registration structure with (red curve) and without (blue curve) the simulated baseline shift (BLS). Dotted lines indicate the displacement threshold for automatic gating. * indicates *p* < 0.05.

The average range of motion within a 95% confidence interval, calculated as the mean plus 2 standard deviations, was approximately 2 mm (1.7–2.1 mm). Therefore, thresholds of ±1.0 mm in the RL, AP, and SI directions were used in the MMRP software to calculate beam delivery efficiency. Delivery efficiency for Patients 3, 4, 6, 7, and 9 was ≤70% due to patient motion (Table 3), which would potentially increase the total beam-on time by more than 30% in clinical scenarios. The average delivery efficiency in these 5 cases was 61.0% ± 6.1%. BLS simulations were conducted for these patients, and the delivery efficiency improved to >80% in four of the five cases (indicated by the first number in red parentheses in Table 3). As shown in Figure 2A, the average delivery efficiency following BLS simulations improved to 80.1% ± 5.2% (*p* = 0.0312). The median number of BLS required for this increased delivery efficiency was three (indicated by the second number in red parentheses in Table 3).

Figure 2B illustrates the case of Patient 3, for whom the delivery efficiency was initially 60%. After three BLS simulations, the delivery efficiency improved to 83%. The top row shows the registration structure centroid position in the LR, AP, and SI directions, respectively, along with the calculated baseline from the BLS simulation. The bottom row shows the resultant displacement values. Without baseline correction (blue curves), the displacement is the initial registration structure position subtracted from the subsequent registration structure positions. With baseline correction (red curves), the displacement is the baseline position subtracted from the registration structure positions. Thus, with the application of BLS simulations, the registration structure remained within the displacement threshold for a substantially greater portion of the time, resulting in a higher duty cycle.

### 3.4. Quality of Simulated Baseline Shift-Corrected Plans

**Figure 3:**
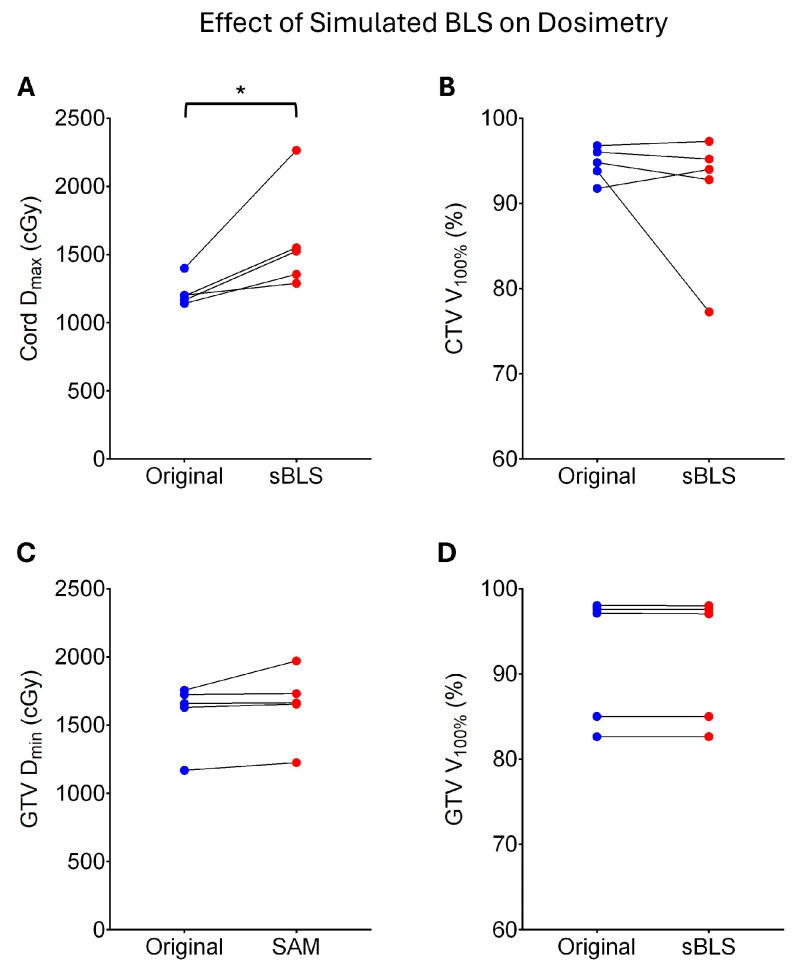
Effects of simulated baseline shifts (sBLS) on dosimetry. Average cord max dose was significantly increased following simulated BLS (**A**). CTV V_100%_ (**B**), GTV D_min_ (**C**), and GTV V_100%_ (**D**) were not significantly impacted. * indicates *p* < 0.05.

Following ATP plan adaptation between the two 3D MR scans, the simulated BLS plans showed significant increases in cord dose (>7%) but remained within the acceptable limit of 14 Gy for Patients 3 and 9. In contrast, the simulated BLS plans for Patients 4, 6, and 7 were not clinically acceptable due to cord overdose, inadequate target coverage, or both, as in the case of Patient 6 (Table 4). Average values for these dose statistics in the original vs. simulated BLS plan were as follows (Figure 3): Cord D_0.01cc_ (1220 cGy ± 103 cGy vs. 1596 cGy ± 390 cGy, p = 0.0213); CTV V_100%_ (94.6% ± 2.0% vs. 91.3% ± 8.0%, *p* = 0.3977); GTV V_100%_ (92.1% ± 7.6% vs. 92.1% ± 7.8%, *p* = 0.2439); GTV D_min_ (1587 cGy ± 240 cGy vs. 1648 cGy ± 269 cGy, *p* = 0.0625).

## 4. Discussion

In addition to providing excellent spinal cord visibility, an MRL equipped with a motion management system could be of considerable benefit for treating spinal tumors with aggressive dose regimens. We determined a candidate registration structure (canal + 3VB) and displacement threshold (±1 mm) for gating from real-time patient motion data, and we found that simulated delivery efficiency was improved by using BLS in most cases, but it may not be effective for patients with persistent large motion (>2 mm) due to low duty cycles.

To identify the most suitable tracking structure, several options were explored. Initially, the spinal cord was chosen; however, tracking of SI motion proved unreliable because of its small size and lack of distinct boundaries in that direction. Moreover, using both the CTV and the spinal cord led to tracking errors during image registration, due to structural similarities with nearby anatomical elements. Ultimately, the optimal choice was found to be the three adjacent vertebral bodies encompassing the spinal canal for robust tracking during radiotherapy procedures. This template strategy is adaptable to different regions of the spine by centering the vertebral window on the lesion, making it feasible for routine use in cervical, thoracic, and lumbar cases. Our findings support the reproducibility of this approach across varied anatomical sites.

In this study, our comparison of real-time cine imaging and 3D T2 MRI for assessing intrafraction motion in spine SBRT demonstrated that the average range of motion measured by cine imaging was 0.2–0.3 mm higher than that determined by pre- and post-cine 3D MRI analysis. This is expected, given the differences between continuous 2D real-time imaging and time-separated 3D image sets. In one of our prior studies, we analyzed table corrections for 78 spine SBRT treatments by using BrainLab ExacTrac for patient setup and intrafraction monitoring, all with the same immobilization setup as this study [32]. Patients were initially positioned by using external marks and aligned with bony anatomy on ExacTrac images. Intrafraction motion averaged 0.6 ± 0.6 mm in the RL direction, 0.3 ± 0.3 mm in AP, 0.5 ± 0.3 mm in SI, and planning target volume (PTV) margins of 1.6 mm for thoracic and lumbar spine SBRT, which is consistent with those measured with 3D MRI in this study. Mesko et al. also reported PTV margins of 1.5–2.0 mm for cervical spine SBRT, based on intrafraction error managed with ExacTrac X-ray images [33]. From our results, we chose a displacement threshold of ±1 mm for simulating the CMM gating, but this is considered only a starting point and may need variation due to patient-specific target/cord geometry and plan complexity. Methods for determining patient-specific thresholds are of great interest for future work.

Intrafraction motion (95%–5%) was 1.0 ± 0.6 mm in RL, 1.0 ± 0.6 mm in AP, and 0.9 ± 0.6 mm in SI for patients with lesions above T5 (patients 1–7, who were immobilized with a mask during treatment). Motion artifacts caused by heart motion, arterial pulsation, and swallowing were observed near T2–T4 spine lesions. For patients with lesions below T5 (patients 8–12, who were immobilized without a mask), intrafraction motion was 0.6 ± 0.4 mm in RL, 0.7 ± 0.5 mm in AP, and 0.5 ± 0.2 mm in SI. Patients with lesions above T5 had slightly larger intrafraction motion in all directions, likely because of cardiac and respiratory movements, although these differences were not statistically significant (Wilcoxon signed rank test *p* > 0.05). Thus, treating lesions above T5 (as in patients 1–7), even with masks used for immobilization, presented additional motion challenges. The analysis of cine images during simulation provides an opportunity to assess whether motion would present challenges during treatment, and whether they could be addressed with beam gating and BLS. Displacement thresholds of ±1.0 mm for gating were derived from the intrafraction motion measurements. This threshold of ±1.0 mm is comparable to spine SBRT IGRT on conventional linacs [34,35]. This is reflective of the high dose gradients that exist between spine targets and the nearby spinal cord. In the context of MR-guided radiotherapy, it is recommended that the gating tolerances be set according to the planning margins to ensure clinical safety and treatment delivery accuracy [36].

A consistent trend was noted in that the average range of motion decreased slightly from the first imaging session to the second imaging session in all three directions. Most patients (except for patient 6) experienced reduced motion during the second simulation, which took place 1–2 weeks after the first. Only 5 of the 23 imaging sessions were significantly shortened (to <15 of the planned 20 min) because of patient discomfort or anxiety (Table 1). Westerhoff et al. reported that anxiety, monitored via heart rate, was most commonly observed before or after the first treatment fraction [37]. Therefore, reducing patient anxiety, e.g., through acoustic noise dampening or patient education, may help improve treatment stability.

Further analysis of the real-time cine data revealed that in 5 of 23 scans, intrafraction motion consistently exceeded the displacement threshold of ±1.0 mm, resulting in calculated delivery efficiency values as low as 55% (Table 3). After the BLS simulation, only one case had a delivery efficiency below 80%—the same case that required the most BLS corrections (*n* = 5). This low efficiency was caused by high-frequency, high-amplitude oscillations of the registration structure, which repeatedly exceeded gating tolerances despite baseline adjustments. Such low efficiency would significantly prolong beam-on time and may be considered suboptimal for clinical implementation without further adaptation. These findings suggest that CMM-based treatment may be inefficient for patients with large target or cord motion (>2 mm), even with BLS. Cine MRI during MR simulation can help identify these patients in advance. In such cases, alternative strategies like loosening the gating tolerances, initiating a completion plan workflow, or implementing full adaptive replanning may be more appropriate.

The criteria used to trigger a BLS in our simulations were designed to best replicate clinical scenarios. However, it is important to note that the simulated BLS applied consistent criteria across all patients, whereas clinical implementation would likely involve patient-specific adjustments in terms of BLS timing, frequency, and tolerance thresholds. Future work will focus on refining BLS trigger criteria to achieve the highest possible delivery efficiency with the fewest BLS events, incorporating patient-specific tolerances and exploring integration with adaptive workflows.

Our simulated BLS dosimetry data demonstrated that although target coverage was generally maintained, this benefit may come at the expense of a higher spinal cord dose. This could be due to the faster segment aperture morphing method not being sufficient to preserve the spinal cord dose in all cases. Even when assuming that the spinal cord moves synchronously with the target in most instances, there remains uncertainty regarding the spinal cord dose in BLS plans, especially when the same number of monitor units and effective depth from the reference plan are applied [26]. An extreme scenario of this was observed in Pt7, where the SAM-based adaptation produced a cord dose > 22 Gy. This would, of course, be caught during the BLS plan review stage prior to reinitiating treatment and would likely require a completion plan, which incorporates more thorough ATP adaptation. A similar observation was made by Dobbelsteen et al., who demonstrated that while BLS improved PTV coverage, it resulted in higher doses to the rectum and bladder compared to the adapted plan produced by the adapt-to-shape workflow in their prostate treatment evaluation study [26]. Further research is crucial to address these uncertainties.

Finally, this study did not investigate the effects of image quality on tracking accuracy. However, image noise is expected to impact tracking performance and, consequently, delivery efficiency. Similarly, the limited pixel resolution of the cine images (0.96 mm) may constrain the minimum achievable displacement threshold for gating. These uncertainties will likely become clearer with broader clinical implementation of the CMM workflow. Although the sample size was limited, this study provides initial insights into the feasibility and dosimetric implications of motion management in MR-guided spine SBRT. Larger-scale validation studies are ongoing to support clinical protocol development.

## 5. Conclusions

This study optimized registration structures and displacement thresholds, contributing to more precise gated treatment. Cine imaging and BLS can improve delivery efficiency in spine SBRT but challenges remain in patients with large, frequent motion. These findings emphasize the need for advanced motion management and patient-specific BLS protocols. While simulated BLS adaptations enhanced delivery efficiency, they also led to increased spinal cord dose in some cases, highlighting trade-offs that require careful patient selection. Ongoing work aims to refine BLS criteria to maximize efficiency while ensuring spinal cord safety.

## Figures and Tables

**Table 1:** Patient and treatment characteristics.

Patient	Target	Immobilization	Rx (GTV/CTV)	1st Cine, min	2nd Cine, min
1	C5	Masked	24/16 (1FX)	20.2	16.2 *
2	T1	Masked	27/21 (3FX)	20.2	14.6 *
3	T2	Masked	18/16 (1FX)	20.2	19.7
4	T2–T3	Masked	24/16 (1FX)	20.2	NA
5	T3	Masked	24/16 (1FX)	20.2	20.2
6	T3	Masked	18/16 (1FX)	20.2	20.2
7	T5	Masked	27/21 (3FX)	20.2	20.2
8	T6	Un-Masked	24/16 (1FX)	20.2	20.2
9	T7	Un-Masked	18/16 (1FX)	5.2 *	20.2
10	T12	Un-Masked	18/16 (1FX)	11.9 *	20.2
11	L1	Un-Masked	18/16 (1FX)	20.2	6.6 *
12	L2	Un-Masked	18/16 (1FX)	11.2 *	20.2

* The scheduled 20-min scan time was incomplete because of patient discomfort. Abbreviations: C, cervical vertebrae; FX, number of fractions; L, lumbar vertebrae; Rx (CTV/GTV), prescribed dose to the clinical target volume (CTV) and gross tumor volume (GTV); T, thoracic vertebrae.

**Table 2:** Range of motion (Max–Min) in cine images from 3 patients based on three different registration structures.

	Patient 3			Patient 4			Patient 12		
	Cord Only	Cord + CTV	Canal + 3VB	Cord Only	Cord + CTV	Canal + 3VB	Cord Only	Cord + CTV	Canal + 3VB
LR, mm	2.0	1.8	1.5	2.5	2.9	2.9	0.9	0.8	0.8
AP, mm	3.4	3.7	3.3	2.2	2.5	2.8	1.5	1.1	1.2
SI, mm	2.3	1.4	1.1	4.5	1.7	1.5	1.6	0.7	0.7

Abbreviations: 3VB, 3 vertebrae adjacent to the canal; AP, anteroposterior; Canal, spinal canal; CTV, clinical target volume; LR, left–right; SI, superior–inferior.

**Table 3:** Delivery efficiency, in %, before and after baseline shift simulations for all 12 patients.

	Pt1	Pt2	Pt3	Pt4	Pt5	Pt6	Pt7	Pt8	Pt9	Pt10	Pt11	Pt12
1st Cine	99.4	99.0	59.5 (82.9, 3)	56.0 (83.0, 3)	99.9	99.2	55.4 (82.9, 4)	94.1	70.0 (83.2, 1)	97.2	99.9	99.3
2nd Cine	99.8	98.0	88.2	NA	99.9	64.0 (71.3, 5)	91.0	88.0	99.8	99.9	99.3	99.9

Abbreviations: Pt, patient. Numbers in red parentheses represent the updated delivery efficiencies after baseline shift simulation and the total number of baseline shifts used.

**Table 4:** Plan quality comparison between reference plans and simulated baseline-shifted plans by using segment aperture morphing (SAM) adaptation.

Dosimetric Changes Following SAM-Based ATP Plan Adaptation															
	Pt 3			Pt 4			Pt 6			Pt 7			Pt 9		
Descriptors of	(0.0 mm LR, 0.0 SI, 1.8 AP)			(1.5 mm LR, 0.5 SI, 2.0 AP)			(0.8 mm LR, 0.5 SI, 0.4 AP)			(0.5 mm LR, 1.5 SI, 2.1 AP)			(1.0 mm LR, 0.8 SI, 1.0 AP)		
Plan Quality	Original	sBLS	Diff	Original	sBLS	Diff	Original	sBLS	Diff	Original	sBLS	Diff	Original	**sBLS**	**Diff**
Cord															
D_0.01cc_, cGy	1140.5	1354	18.7%	1164.5	1524.5	30.9%	1195.5	1550.5	29.7%	1398.5	2264.5	61.9%	1202	1288.5	7.2%
CTV															
V_100_, cGy	91.75	93.98	2.2%	96.78	97.28	0.5%	93.8	77.26	−16.5%	94.78	92.78	−2.0%	96.02	95.2	−0.8%
GTV															
D_min_, cGy	1723.3	1730.9	0.4%	1167.8	1224.6	4.9%	1657.6	1662.8	0.3%	1755.3	1970.2	12.2%	1629.4	1653.3	1.5%
V_100_, %	97.13	97.04	−0.1%	85	84.99	0.0%	98.02	98	0.0%	82.63	82.64	0.0%	97.58	97.58	0.0%

Numbers in red indicate a violation of clinical goals (cord D_0.01cc_ > 1400 cGy or CTV V_100_ < 80%). Abbreviations: AP, anteroposterior; ATP, adapt-to-position; CTV, clinical target volume; GTV, gross tumor volume; LR, left–right; Pt, patient; sBLS, simulated baseline shift; SI, superior–inferior.

## Data Availability

Data are available upon request.

## Abbreviations

The following abbreviations are used in this manuscript:

SBRT	stereotactic body radiation therapy
MR	magnetic resonance
Linac	linear accelerator
MRL	MR linac
CMM	comprehensive motion management
MMRP	motion management research package
GTV	gross tumor volume
CTV	clinical target volume
PTV	planning target volume
SAM	segment aperture morphing
ATP	adapt-to-position
ATS	adapt-to-shape
VB	vertebral body
RL	right-left
AP	anterior–posterior
SI	superior–inferior
BLS	baseline shift
sBLS	simulated BLS
